# Baicalin Ameliorates Liver Injury Induced by Chronic plus Binge Ethanol Feeding by Modulating Oxidative Stress and Inflammation via CYP2E1 and NRF2 in Mice

**DOI:** 10.1155/2017/4820414

**Published:** 2017-08-16

**Authors:** Ping He, Yafeng Wu, Jianchao Shun, Yaodong Liang, Mingliang Cheng, Yuping Wang

**Affiliations:** ^1^Department of Clinical Microbiology and Immunology, Affiliated Hospital of Guizhou Medical University, Guizhou Medical University, Yunyan District, Guiyang City, Guizhou Province 550004, China; ^2^Guizhou Provincial Center for Clinical Laboratory, No. 83 Zhongshandong Road, Guiyang City, China; ^3^Public Health Treatment Center of Guiyang, No. 6 Daying Road, Guiyang City, China; ^4^Department of Infectious Diseases, Affiliated Hospital of Guizhou Medical University, Guiyang City, Guizhou Province 550004, China

## Abstract

Alcoholic liver injury leads to serious complication including death. The potential role of baicalin at the transcription level in mice model of alcohol injury is not known yet. In this study, we examined the effect of baicalin against chronic plus binge ethanol model in mice and understanding the mechanism of protection. Liver function, histology, steatosis, inflammation, NF-*κ*B activity, oxidative stress sources, nuclear translocation of NRF2 transcription factor, and cell death were assessed. Treatment with baicalin ameliorated ethanol-induced oxidative stress, inflammation, and cell death. Baicalin attenuated ethanol-induced proinflammatory molecules such as TNF-*α*, IL-1*β*, MIP-2, and MCP-1 and reversed redox-sensitive transcription factor NF-*κ*B activation. Baicalin also modulated Kupffer cell activation in vitro. Baicalin inhibited ethanol-induced expression of reactive oxygen species (ROS) generating enzymes NOX2, p67phox, xanthine oxidase, and iNOS in addition to CYP2E1 activities. Baicalin also enhanced ethanol-induced NRF2 nuclear translocation and increased downstream target gene HO-1 as antioxidant defense. Finally, baicalin reduced significant apoptotic and necrotic cell death. Our study suggests that baicalin ameliorates chronic plus binge ethanol-induced liver injury involving molecular crosstalk of multiple pathways at the transcriptional level and through upregulation of antioxidant defense mechanism.

## 1. Introduction

Alcoholic liver disease (ALD) in human covers a wide spectrum of abnormality in liver such as steatosis, steatohepatitis, and cirrhosis. Some of cirrhosis patients also lead to hepatocellular carcinoma [1]. It is difficult to replicate similar model in mice due to their alcohol-metabolizing pattern and specific physiologic parameter. One of the optimized mice model similar to ALD in human is developed by Dr. Gao's group and it is a chronic plus binge alcohol model [2]. In this model, alcohol induced steatohepatitis and cell death which leads to significant increase in serum AST and ALT [3, 4]. Inflammation and oxidative stress in the liver is considered a major player in chronic plus binge alcohol feeding model [5, 6]. Modulation of inflammation and oxidative stress or its downstream targets associated with cell death is used to ameliorate the liver damage of alcohol. Plant-derived flavonoids are well-known for its antioxidant and anti-inflammatory properties [7, 8]. Flavonoids were used in many oxidative tissue injury models in mice, and role of different transcription factor including NRF2 has been implicated [9–11].

Baicalin, a flavonoid, is present in Chinese medicinal plant *Scutellaria baicalensis* Georgi. Baicalin have demonstrated hepatoprotective effects by modulating oxidative stress, inflammation, and fibrosis. Baicalin ameliorates diet-induced obesity through AMPK pathway in mice [12]. In a T cell-mediated liver injury model, baicalin demonstrates anti-inflammatory properties [13]. Liver fibrosis is mitigated by baicalin through PPAR*γ* and TGF*β*1 in a rat model of carbon tetrachloride-induced liver injury [14]. In a murine orthotopic hepatocellular carcinoma model, baicalin modulates macrophage polarization [15]. However, the role of baicalin as antioxidant and anti-inflammatory and the role of specific correlated transcription factors are not known till now.

Here, we have demonstrated that baicalin protects against alcoholic liver injury in mice by reducing inflammation through NF-*κ*B and ameliorates oxidative stress through CYP2E1 and NRF2 transcription factor.

## 2. Materials and Methods

### 2.1. Animal Study

Mice were purchased from the Animal Center of Guizhou Medical University (approval number SCXK (Guizhou) 2002-0001, Guiyang, China). All animal experiments were approved by the Guizhou Medical University Animal Care and Use Committee. Twelve-week-old male and female mice with weight over 22 g were subjected to either pair-fed or alcohol feeding protocol according to earlier publication [4]. Briefly, mice were pair-fed with an isocaloric control diet (pair-fed) or ethanol Lieber-DeCarli diet (EtOH) for 10 days. On day 11, ethanol and pair-fed mice were provided gavage with a single dose of ethanol (5 g/kg b.w.) or isocaloric dextrin-maltose, respectively, and sacrificed 9 hours later. Baicalin (Sigma-Aldrich, USA) was dissolved in vehicle (dimethyl sulfoxide (DMSO) : water in 1 : 9 ratio) and injected i.p. (200 mg/kg/day) for 11 days. Vehicle solution was administered in control experiments. Serum alanine aminotransferase (ALT) and aspartate aminotransferase (AST) levels were determined (Zhongsheng BeiKong Biological Technology Limited Corporation, Beijing, China).

### 2.2. Histopathology

Liver specimens were embedded in paraffin. Paraffin-embedded tissues were deparaffinized and were then subjected to hematoxylin and eosin (H&E) staining. The specific staining was visualized, and images were acquired using a microscope.

### 2.3. NF-*κ*B Activity Assay

Binding of NF-*κ*B p65 subunit to the NF-*κ*B binding consensus sequence 5′-GGGACTTTCC-3′ was measured with the ELISA-based TransAm NF-*κ*B kit (Active Motif, USA) according to manufacturer's instruction. The kit contain 96-well microtiter plates coated with an oligonucleotide containing the NF-*κ*B binding consensus sequence. The active form of p65 subunit was detected using antibody specific for an antigen when the subunit is activated and bound to its target DNA.

### 2.4. Fluorescence Imaging

Sections from frozen livers were incubated with paraformaldehyde (4%) after drying in air for 1 hour. Following fixation, tissue sections were incubated with 0.1 *μ*M of Nile Red in phosphate-buffered saline and examined by confocal microscopy.

### 2.5. TUNEL Staining

Apoptosis in liver sections was detected by terminal deoxynucleotidyl transferase-mediated dUTP nick-end labeling (TUNEL) using the In Situ Cell Death Detection Kit (Roche, Germany) according to manufacturer's instruction.

### 2.6. Hepatic Triglyceride

Triglycerides from liver lysates were extracted with a 2 : 1 chloroform : methanol mixture and quantified using the EnzyChrom Triglyceride Assay Kit (BioAssays Systems, Hayward, CA).

### 2.7. PARP Activity

PARP activity was detected using colorimetric PARP Assay Kit (Amyjet Scientific Inc., Wuhan, China) according to the manufacturer's instructions. Data were expressed as fold change of control values.

### 2.8. DNA Fragmentation

The extent of hepatic DNA fragmentation was determined using an enzyme-linked immunosorbent assay (ELISA) kit (Boehringer Mannheim AG, Switzerland) according to the manufacturer's instructions.

### 2.9. Real-Time Reverse Transcription Polymerase Chain Reaction

Total RNA was extracted from frozen liver samples using the RNeasy Mini Kit (Qiagen Inc.) according to the manufacturer's protocol. RNA (2 *μ*g) for each analyzed sample was reverse-transcribed in a final volume of 25 *μ*l using M-MLV Reverse Transcriptase (Promega). Quantitative real-time PCR reaction was performed in a final volume of 20 *μ*l containing 1 *μ*l of cDNA (1 : 10 dilution) and 500 nM of primers (all primers were obtained from Qiagen) and SYBR Green PCR premix (Takara) according to the manufacturer's instructions. The expression levels of target genes were normalized to the expression of beta-actin and quantified based on the comparative cycle threshold Ct method (2^−ΔΔCt^).

### 2.10. Western Blot Analysis

Liver tissues were lysed in 200 *μ*l RIPA buffer (0.05 M Tris-HCl pH 7.4, 0.15 M NaCl, 0.25% deoxycholic acid, 1% Nonidet P-40, 1 mM EDTA, 1 mM phenylmethylsulfonyl fluoride, 10 *μ*g/ml aprotinin, and 10 *μ*g/ml leupeptin). The nuclear lysates were harvested with NucBuster Protein Extraction Kit (Novagen, Germany) according to manufacturer's instructions. Protein concentrations were measured using the bicinchoninic acid (BCA) protein assay (Pierce, USA). Proteins were analyzed by SDS-PAGE and transferred to nitrocellulose membranes. The blots were probed with the following primary antibodies: NRF2 (Santa Cruz Biotechnology, USA) and histone H3 (Cell Signaling Technology, USA), followed by incubation with species-matched secondary antibodies. The bands were detected using enhanced chemiluminescence (Pierce, USA).

### 2.11. CYP2E1 Activity

Liver microsomal fractions were isolated using Microsome Isolation Kit (Biovision, USA) according to manufacturer's manual. CYP2E1 activities from microsomal fractions were determined as previously described [16]. In briefly, CYP2E1 activity was determined by the hydroxylation of aniline into blue color p-aminophenol using plate reader at 630 nm and expressed as nmol/min/mg protein.

### 2.12. Kupffer Cell Isolation from Mice and In Vitro Studies

Kupffer cells were isolated as described earlier [17]. Isolated Kupffer cells were incubated with saline or LPS (1 *μ*g/ml) for 6 hours with or without baicalin (50 *μ*g/ml). Cell culture supernatants were analyzed for quantification of TNF*α* using ELISA kit (Abcam, China).

### 2.13. Statistical Analysis

All the values are represented as mean ± SEM. Statistical analysis of the data was performed by analysis of variance (ANOVA) for multiple comparisons. The analysis was conducted using GraphPad Prism software. *P* < 0.05 was considered statistically significant with *n* = 6 or 4/group.

## 3. Results

### 3.1. Baicalin Treatment Ameliorates Chronic plus Binge Ethanol-Induced Liver Injury

**Figure 1:**
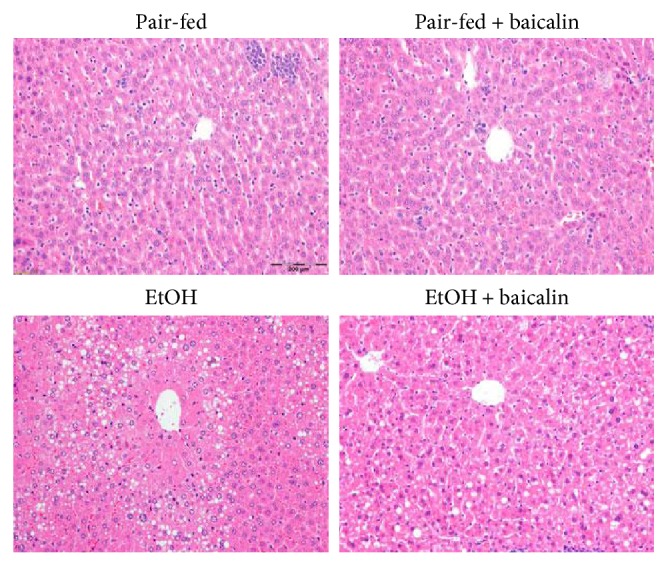
Baicalin treatment prevents histopathological liver damage in chronic-binge ethanol-fed mice. Representative H&E staining of liver sections.

**Figure 2:**
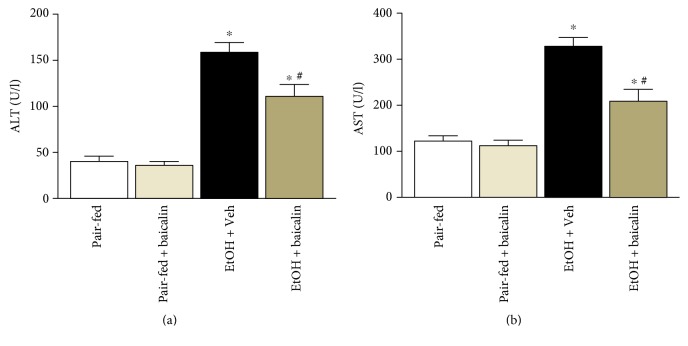
Baicalin treatment prevents chronic-binge ethanol-induced liver injury. Serum ALT and AST level values represent means ± SEM and *n* = 6/group. ^∗^*P* < 0.05 versus pair-fed group, ^#^*P* < 0.05 versus EtOH group.

Mice were fed with liquid diet containing 5% ethanol, and a single binge 9 hour before sacrifice induced significant liver injury as shown by hematoxylin and eosin staining (Figure 1). Liver injury was also evident from increased serum transaminases ALT (3.9-fold) and AST (2.7-fold). Baicalin treatment at 200 mg/kg/day dose reduced both ALT and AST at significant level 30.1% and 32.9%, respectively (Figure 2). There was no difference between pair-fed groups of vehicle and baicalin. Baicalin dose was in consistent with earlier publications [12, 13, 18].

### 3.2. Baicalin Treatment Attenuates Chronic plus Binge Ethanol-Induced Liver Injury and Steatosis

**Figure 3:**
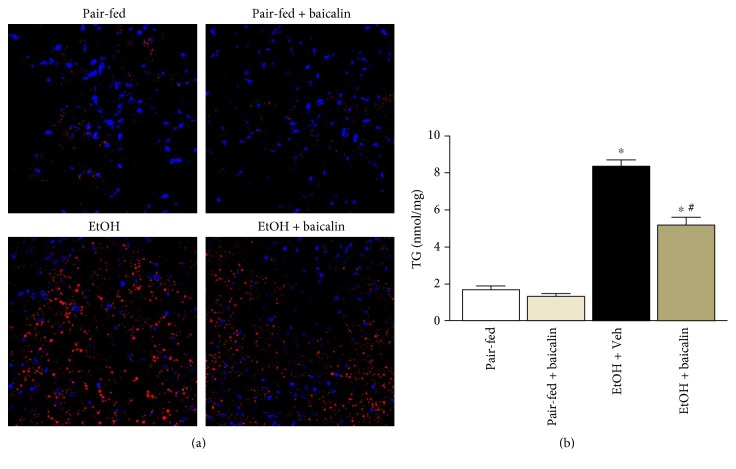
Baicalin treatment ameliorates chronic-binge ethanol-induced liver steatosis (a) Representative Nile red staining of frozen liver sections and (b) liver triglyceride content. Values represent means ± SEM and *n* = 6/group. ^∗^*P* < 0.05 versus pair-fed group, ^#^*P* < 0.05 versus EtOH group.

Ethanol feeding led to increased fat deposition in the liver as evident by Nile red staining (Figure 3(a)). Quantitative determination of triglyceride content in the liver also demonstrated a 4.7-fold increase by ethanol feeding (Figure 3(b)). Baicalin attenuated Nile red staining of oil droplets and also reduced 38% of liver triglyceride content from ethanol-fed mice. No changes were observed between pair-fed groups.

### 3.3. Baicalin Attenuates Chronic-Binge Ethanol-Induced Inflammatory Cytokines and Its Regulator NF-*κ*B

**Figure 4:**
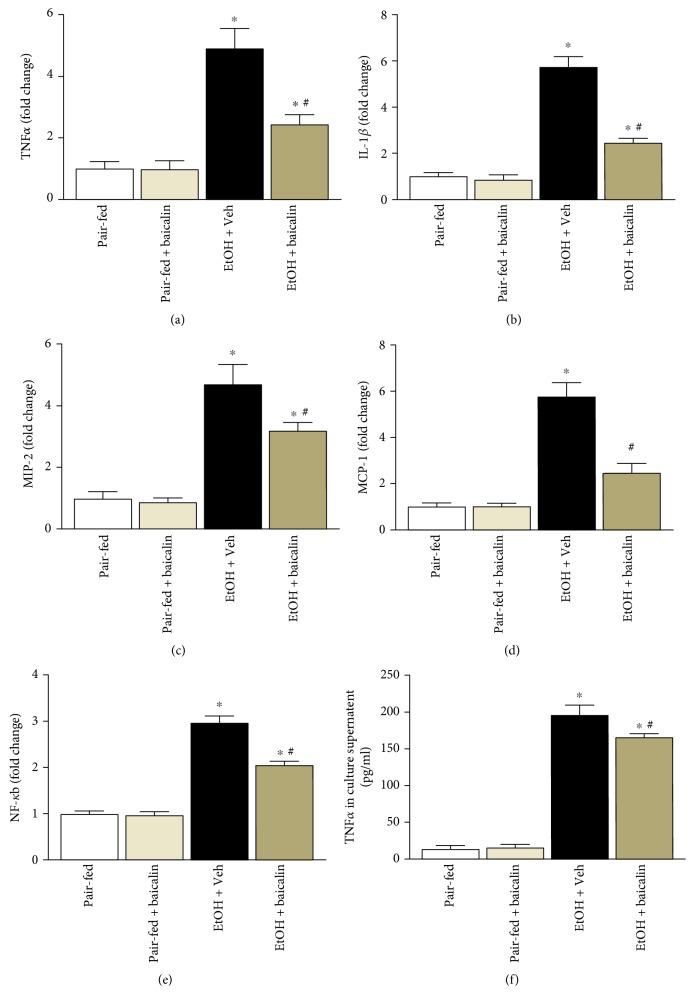
Baicalin treatment ameliorates against chronic-binge ethanol-induced liver injury by attenuating liver inflammation. Liver mRNA levels determined by real-time PCR of TNF-*α* (a), IL-1*β* (b), MIP-2 (c), and MCP-1 (d). The results are expressed as fold change relative to the pair-fed group. Values represent means ± SEM and *n* = 6/group. ^∗^*P* < 0.05 versus pair-fed group, ^#^*P* < 0.05 versus EtOH group. (e) Transcriptional activity of NF-*κ*B were determined and represented as fold change. Values represent means ± SEM and *n* = 6/group. ^∗^*P* < 0.05 versus pair-fed group, ^#^*P* < 0.05 versus EtOH group. (f) TNF-*α* in Kupffer cell culture supernatant determined by ELISA assay. Values represent means ± SEM and *n* = 4/group. ^∗^*P* < 0.05 versus control, ^#^*P* < 0.05 versus LPS + Veh group.

Chronic plus binge ethanol feeding induced inflammatory cytokines TNF*α*, IL-1*β*, MIP-2, and MCP-1 to 4.9-, 5.7-, 4.7-, and 5.8-fold, respectively (Figures 4(a), 4(b), 4(c), and 4(d)). Baicalin treatment reduced TNF*α*, IL-1*β*, MIP-2, and MCP-1 cytokine/chemokine levels up to 49%, 57%, 31%, and 57% respectively. NF-*κ*B controls the global proinflammatory response, and we investigated its role in the liver. Increased NF-*κ*B activity in chronic plus binge ethanol feeding mice liver clearly demonstrated its role (Figure 4(e)).

Kupffer cells in the liver plays a critical role in liver inflammation. We also examined the effect of baicalin on Kupffer cell activation by endotoxin lipopolysaccharide (LPS, 1 *μ*g/ml, 6 hours). Baicalin treatment (50 *μ*g/ml) attenuated LPS-induced TNF*α* secretion from Kupffer cells (Figure 4(f)).

### 3.4. Baicalin Attenuates Ethanol-Induced ROS-Generating Sources

**Figure 5:**
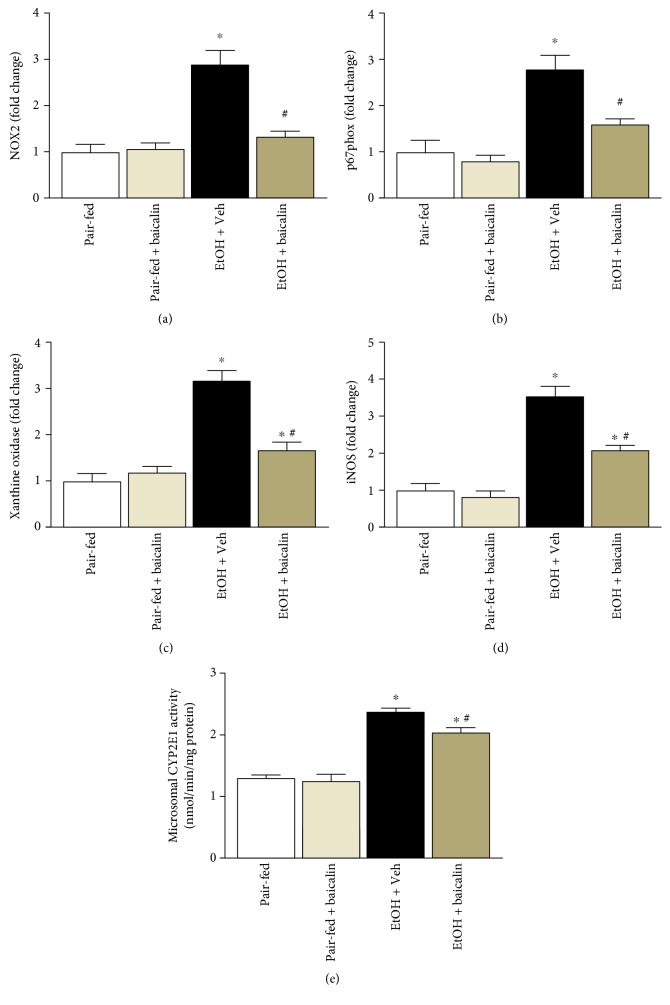
Baicalin treatment reduces mRNA expression of ROS-generating enzymes in the liver tissues. Hepatic mRNA of NOX2 (a), p67phox (b), xanthine oxidase (c), and iNOS (d) were analyzed by real-time PCR. The results are expressed as fold change relative to the pair-fed group. Values represent means ± SEM and *n* = 6/group. ^∗^*P* < 0.05 versus pair-fed group, ^#^*P* < 0.05 versus EtOH group. (e) Microsomal CYP2E1 activities among various groups. Values represent means ± SEM and *n* = 4/group. ^∗^*P* < 0.05 versus pair-fed group, ^#^*P* < 0.05 versus EtOH group.

Ethanol-induced liver injury is known to be associated with increased oxidative and nitrative stress. We observed that ethanol feeding induced mRNA expression of ROS-generating enzymes NOX2 (2.9-fold), p67phox (2.8-fold), xanthine oxidase (3.2-fold), and iNOS (3.6-fold) (Figures 5(a), 5(b), 5(c), and 5(d)). Baicalin reduced ethanol feeding-induced ROS-generating enzymes NOX2, p67phox, xanthine oxidase, and iNOS to 54%, 42%, 53%, and 40%, respectively.

We also determined CYP2E1 activity in microsomal fractions of the liver from all groups. CYP2E1 activity was increased in ethanol feeding mice groups, and baicalin treatment attenuated ethanol feeding-associated increase of CYP2E1 activity (Figure 5(e)).

### 3.5. Baicalin Attenuates Ethanol-Induced Cell Death in Liver

**Figure 6:**
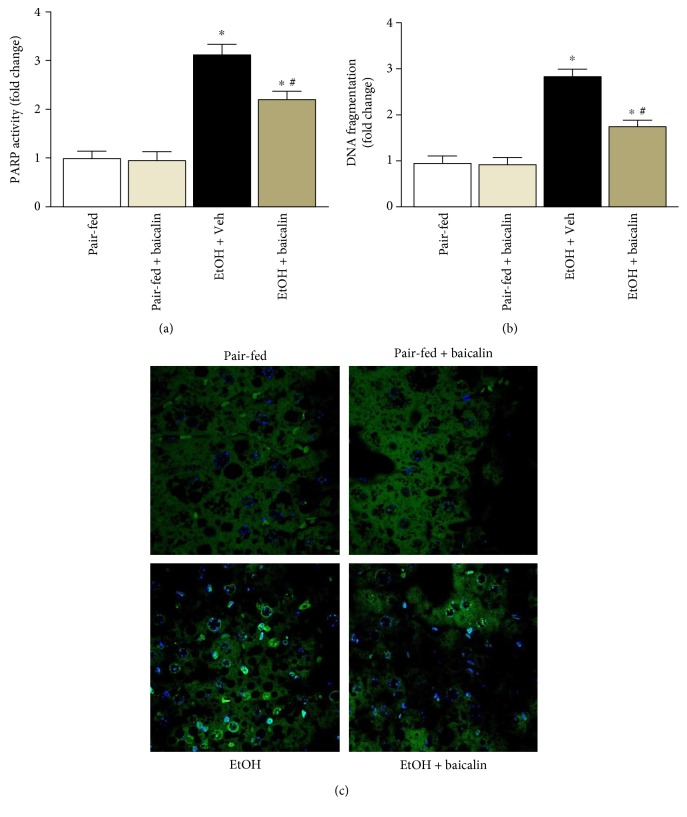
Baicalin prevents chronic-binge ethanol-induced liver injury by attenuating necrotic/apoptotic cell death. (a) Necrotic/apoptotic cell death pathway marker PARP activity was determined quantitatively and represented as fold change. Values represent means ± SEM and *n* = 6/group. ^∗^*P* < 0.05 versus pair-fed group, ^#^*P* < 0.05 versus EtOH group. Apoptotic markers DNA fragmentation (b) and representative TUNEL images (c) were provided. Values represent means ± SEM and *n* = 6/group. ^∗^*P* < 0.05 versus pair-fed group, ^#^*P* < 0.05 versus EtOH group.

Chronic plus binge ethanol feeding leads to both necrotic and apoptotic cell death in the liver. We determined PARP activity and DNA fragmentation by quantitative method. Ethanol induced PARP activity and DNA fragmentation to 3.1- and 2.86-fold, respectively (Figures 6(a) and 6(b)). Baicalin treatment led to 29% and 38% decrease of ethanol feeding-induced PARP activity and DNA fragmentation, respectively. We also did qualitative determination by TUNEL staining. In consistent to DNA fragmentation data, ethanol feeding mice have higher TUNEL-positive cells compared to pair-fed controls and baicalin treatment reduced TUNEL-positive cells in the liver (Figure 6(c)).

### 3.6. Baicalin Enhances Nuclear Translocation of NRF2 and Its Target Genes

**Figure 7:**
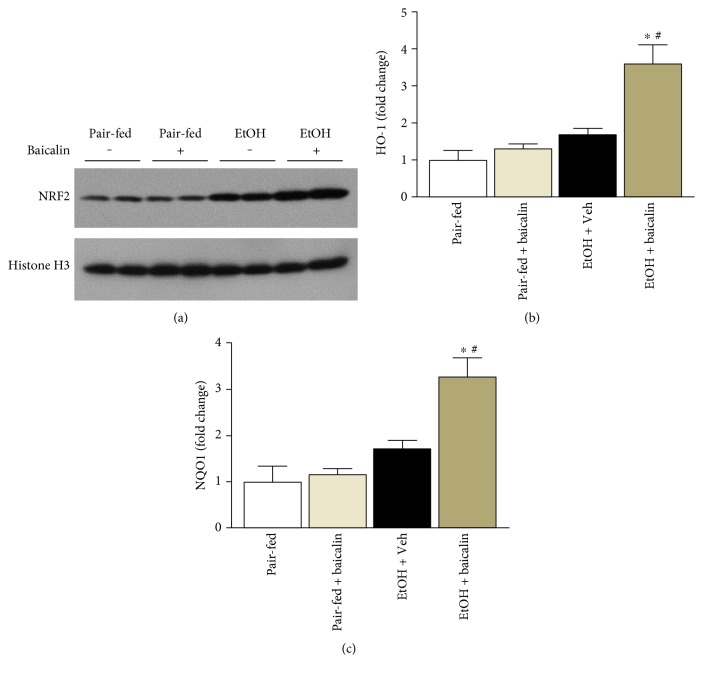
Baicalin prevents chronic-binge ethanol-induced liver injury by modulating NRF2 and its target genes HO-1 and NQO1. (a) Western blot analyses of nuclear extracts of NRF2 along with loading control histone H3. Hepatic mRNA of HO-1 and NQO1 was analyzed by real-time PCR. The results are expressed as fold change relative to the pair-fed group. Values represent means ± SEM and *n* = 6/group. ^∗^*P* < 0.05 versus pair-fed group, ^#^*P* < 0.05 versus EtOH group.

We determined the nuclear translocation of NRF2 in liver tissues. There was increased nuclear accumulation of NRF2 in the liver from ethanol-fed mice and baicalin pair-fed mice. Treatment with baicalin further increased the nuclear accumulation of NRF2 significantly (Figure 7(a)). To verify further the transcriptional response of NRF2, we performed real-time PCR analyses of two targets HO-1 and NQO1. In consistent with nuclear localization of NRF2, baicalin enhanced mRNA level of HO-1 and NQO1 in ethanol-fed mice significantly (Figure 7(b)).

## 4. Discussion

Alcoholic liver disease is associated with steatosis, inflammation, oxidative stress, and cell death involving perivenular hepatocytes to periportal hepatocytes depending on severity [19]. In mouse model of chronic plus ethanol binge, all those three parameters are increased and are similar to human pathology [2, 20]. Here, we demonstrated that baicalin ameliorates chronic plus binge ethanol-induced steatosis, inflammation, oxidative stress, and cell death in mice. Increased production of reactive oxygen species and immediate response to antioxidant defense have been reported in liver injury models [21]. Here, we also demonstrated the protective role of baicalin-induced NRF2 antioxidant defense in response to oxidative stress induced by ethanol.

Liver steatosis is a basic hallmark in alcoholic liver disease, and alcohol-induced metabolic alterations lead to increased fatty acid synthesis and decrease fat metabolism [22]. Circulating lipids are also associated with ALD and proposed as biomarkers in risk assessment [23]. We have demonstrated increased fat deposition in chronic plus ethanol-fed mice liver by Nile red staining and triglyceride content. Both parameters were significantly decreased by baicalin treatment in this study. In a diet-induced obesity model of mice, baicalin dose-dependently decrease hepatic steatosis by inhibiting AMPK/ACC pathway [12]. Baicalin also attenuates high-fat diet-induced insulin resistance and ectopic fat storage in the skeletal muscle, by modulating the protein kinase B/glycogen synthase kinase 3 beta pathways [24].

Inflammation plays a crucial role in the initiation and progression of alcoholic liver disease [25]. Chronic plus binge ethanol feeding induces a series of proinflammatory cytokines and chemokines [4]. We also observed increase in proinflammatory cytokines TNF*α*, IL-1*β*, MIP-2, and MCP-1 in ethanol-fed mice. Baicalin ameliorated all ethanol-induced cytokines. Our data is in consistent with earlier studies in rat model of steatohepatitis [18]. Baicalin is well-known for its anti-inflammatory properties in many disease models in rodent [26–28]. Transcription factor NF-*κ*B stays in cytoplasm as inactive form and translocate to nucleus in response to ethanol [29]. Based on literature, significant crosstalk among NF-*κ*B, inflammation, and oxidative stress exists and the role of NF-*κ*B as key inflammation modulator has been reported [30]. NF-*κ*B activation relates to induction of proinflammatory cytokines and other ROS-generating genes like iNOS [31]. Baicalin reversed ethanol-induced NF-*κ*B activity significantly and thus regulates inflammatory response in this study. Baiclain has been reported to modulate NF-*κ*B in earlier studies [27, 32, 33]. In the present study, baicalin treatment significantly attenuated ethanol-induced release of proinflammatory cytokines, reduced NF-*κ*B activity, and thus inhibited liver inflammation.

Kupffer cell plays critical role in alcoholic and nonalcoholic steatohepatitis [17, 34]. We also observed baicalin-modulated endotoxin-mediated Kupffer cell activation *in vitro*. Baicalin-mediated modulation of Kupffer cell is also reported in other liver injuries [14, 35].

Oxidative stress and associated cell death is key factor in chronic plus binge ethanol feeding. Intervention of cell death pathway such as PARP has been successful in preventing ethanol-induced liver dysfunction and other liver injuries [17, 36]. Excess production of ROS leads to damage of basic molecules in hepatocytes like DNA, proteins, and lipids, which exhibit necrotic and apoptotic cell death [37]. In the present study, we observed four key sources for production of ROS such as NOX2, p67phox, xanthine oxidase, and iNOS in chronic plus binge ethanol feeding and all of them were attenuated by baicalin treatment. Thus, baicalin reduced oxidative/nitrative stress and associated necrotic/apoptotic cell death pathway such as PARP. However, our study does not exclude other sources of ROS including mitochondria in contributing ethanol-induced cell death.

One of key pathways in alcoholic steatohepatitis appears to be the induction of CYP2E1 by ethanol [38]. We also observed similar increase in CYP2E1 activities in chronic plus binge ethanol feeding mice. Baicalin attenuated CYP2E1 activities in microsomal fraction and thus reducing oxidative stress. In previous study, hepatoprotective effect of baicalin has been attributed to modulation of CYP2E1 activities in acetaminophen-mediated liver injury [39].

NRF2, a transcription factor, is a well-known defense against oxidative/nitrative stress in various tissue injury models. Previous publications have demonstrated the upregulation of NRF2 as a strategy to ameliorate alcohol-induced liver injury [40, 41]. Nuclear translocation of NRF2 and the transcriptional activation of its target genes including HO-1 are important for hepatocyte anti-oxidant defense. In our study, we observed baicalin increased nuclear localization of NRF2 in chronic alcohol plus binge ethanol-fed mice. In addition to that, NRF2 target genes HO-1 and NQO1 were also increased suggesting transcriptionally activation of NRF2 targets. Ethanol feeding also increases nuclear NRF2 due to other oxidative stress stimuli. However, baicalin treatment further enhanced nuclear localization of NRF2 compared to ethanol-fed mice liver. Modulation of nuclear localization of NRF2 in oxidative injury by administration of baicalin analog baicalein has been reported earlier in HepG2 cells and cisplatin nephrotoxicity [42, 43]. After administration of baicalein, it modified mainly to its glucuronide analog baicalin in the blood stream with significantly stability (half-life of 11.8 hours) [44, 45]. One of the important findings is that the absence of NRF2 can exacerbate NF-*κ*B activity leading to increased cytokine production, whereas NF-*κ*B can modulate NRF2 transcription and activity. Pharmacological and genetic studies suggest that there is functional crosstalk between these two important pathways at the molecular level [46].

## 5. Conclusions

The present study supports the hypothesis that baicalin is a promising molecule against ethanol-induced liver steatosis, inflammation, and injury. The protective effect of baicalin in chronic plus binge ethanol-induced liver damage is a combination of antioxidant, anti-inflammation, and modulation of fatty acid metabolism. Baicalin modulates both NF-*κ*B and NRF2 transcription factors in addition to CYP2E1 activity which are major regulators of inflammation and antioxidant defense in ethanol-induced liver injury.
